# Determinants of persistent asthma in young adults

**DOI:** 10.1080/20018525.2018.1478593

**Published:** 2018-06-05

**Authors:** Lisbet Krogh Traulsen, Anders Halling, Jesper Bælum, Jesper Rømhild Davidsen, Martin Miller, Øyvind Omland, David Sherson, Torben Sigsgaard, Trine Thilsing, Gert Thomsen, Lars Rauff Skadhauge

**Affiliations:** a Department of Occupational Medicine, Hospital of South West Jutland, Esbjerg, Denmark; b Institute of Regional Health Services Research, Faculty of Health Sciences, University of Southern Denmark, Odense, Denmark; c Center for Primary Health Care Research, Department of Clinical Sciences, Malmö, Lund University, Malmö, Sweden; d Department of Occupational and Environmental Medicine, Odense University Hospital, Odense, Denmark; e Research Unit of General Practice, Department of Public Health, University of Southern Denmark, Odense, Denmark; f Department of Respiratory Medicine, Odense University Hospital, Odense, Denmark; g Institute of Occupational and Environmental Medicine, University of Birmingham, Birmingham, UK; h Department of Occupational Medicine, Danish Ramazzini Centre, Aalborg University Hospital, Aalborg, Denmark; i Department of Public Health, Danish Ramazzini Centre, University of Aarhus, Aarhus, Denmark; j Research Unit for Occupational and Environmental Medicine, Institute of Clinical Research, University of Southern Denmark, Odense, Denmark

**Keywords:** Asthma, prognosis, age of onset, asthma score

## Abstract

**Objective:** The aim of the study was to evaluate determinants for the prognosis of asthma in a population-based cohort of young adults.

**Design:** The study was a nine-year clinical follow up of 239 asthmatic subjects from an enriched population-based sample of 1,191 young adults, aged 20–44 years, who participated in an interviewer-administered questionnaire and clinical examination at baseline in 2003–2006. From the interview, an asthma score was generated as the simple sum of affirmative answers to five main asthma-like symptoms in order to analyse symptoms of asthma as a continuum. The clinical examination comprised spirometry, bronchial challenge or bronchodilation, and skin prick test.

**Results**: Among the 239 individuals with asthma at baseline 164 (69%) had persistent asthma at follow up, while 68 (28%) achieved remission of asthma and seven (3%) were diagnosed with COPD solely. Determinants for persistent asthma were use of medication for breathing within the last 12 months: Short-acting beta-adrenoceptor agonists (SABA) only (OR 3.39; 95%CI: 1.47–7.82) and inhaled corticosteroids (ICS) and/or long-acting beta-adrenoceptor agonists (LABA) (8.95; 3.87–20.69). Stratified by age of onset determinants for persistence in individuals with early-onset asthma (age less than 16 years) were FEV₁ below predicted (7.12; 1.61–31.50), asthma score at baseline (2.06; 1.15–3.68) and use of ICS and/or LABA within 12 months (9.87; 1.95–49.98). In individuals with late-onset asthma the determinant was use of ICS and/or LABA within 12 months (6.84; 2.09–22.37).

**Conclusions**: Pulmonary function below predicted, severity of disease expressed by asthma score and use of ICS and/or LABA were all determinants for persistent early-onset asthma, whereas only use of ICS and/or LABA was a determinant in late-onset asthma. A high asthma score indicated insufficient disease control in a substantial proportion of these young adults.

## Introduction

Asthma is a common complex respiratory disorder with various overlapping phenotypes [1,2]. Common features include fluctuating respiratory symptoms associated with variable airflow limitation and bronchial hyperresponsiveness (BHR) due to inflammation of the airways. The age of asthma onset is an important factor for dividing the phenotypes and a major determinant of the prognosis [3–5], but the prognosis for adult-onset asthma is only sparsely documented [6]. In a prospective study of individuals with adult-onset asthma higher age, higher body mass index (BMI) and low lung function were associated with greater asthma severity, while non-sensitisation and a normal lung function were predictors for remission [7]. A review has shown that adult-onset asthma has a worse prognosis and a lower response to standard asthma treatment than childhood-onset asthma [8]. In a 12-year follow-up study of adult-onset asthma elevated BMI at baseline, smoking and current allergic or persistent rhinitis predicted uncontrolled asthma, and elevated blood eosinophils and good lung function (FEV1) at baseline protected from uncontrolled asthma [9]. Remission rates vary widely due to varying definitions of asthma and observation time but generally early-onset asthma has a substantially higher remission rate than late-onset asthma [10].

Despite reported remission of asthma the disease is usually considered as a treatable, but not curable disease once present [11]. The understanding of determinants that affect the course of diagnosed asthma, e.g. avoidance of environmental or occupational exposures [12], is therefore important for tertiary prevention, since asthma persistence is associated with frequent and severe symptoms with development of impaired lung function [13].

In a recent publication, we have reported risk factors for incident asthma in a cohort of young adults [14]. The aim of the present study was to evaluate determinants for the prognosis of asthma in the same cohort.

## Methods

The present study was a 9-year clinical follow up of 239 individuals with asthma from an enriched population-based sample of 1,191 young adults who participated in an interviewer-administered questionnaire and clinical examination at baseline in 2003–2006, the RAV-study (Risk Factors for Asthma in Adults). The protocol was based on the European Community Respiratory Health Survey II (ECRHS II) [15] and the baseline study has been reported elsewhere [16]. In brief, the baseline study population comprised a random sample of 10,000 individuals, aged 20–44 years and standardised by sex and age. Among 7,271 (73%) individuals who answered a screening questionnaire (Phase 1), a random sample corresponding to 20% of the study population plus a complementary symptom group of individuals reporting respiratory symptoms were invited to an interview and clinical examination (Phase 2). Of 1,191 subjects who participated in the clinical examination at baseline 424 had asthma. A total of 742 (62%) individuals were re-examined at follow up in 2012–2014 leaving 239 subjects with asthma at baseline for further analysis.

Ethical approval for the study was obtained by The Regional Scientific Ethical Committee for Southern Denmark and written informed consent was obtained from all participants.

### Interview

The interview at baseline and follow up was a slightly modified electronic ECRHS main questionnaire performed in connection to the clinical examination by skilled interviewers trained in standardised interview technique. The interview comprised items on asthma history, asthma-like symptoms, medication, smoking habits, education and occupation.

### Clinical examination

The clinical examination at baseline comprised a spirometry using a MicroLoop Spirometer (Micro Medical, Rochester, UK) and a skin prick test (SPT). The spirometry was followed by a methacholine challenge test using a Mefar MB3 dosimeter (Mefar, Bovezzo, Italy) or bronchodilation by inhalation of Terbutalin, 1.5 mg if forced expiratory volume in 1 s (FEV₁) was <70% of predicted or <1.5 l. The SPT comprised a panel of 13 commercially available inhalation allergens from ALK-Abelló, Gentofte, Denmark.

The spirometry at follow up was carried out by using an EasyOne Spirometer (ndd Medical Technologies, Andover, MA, USA) followed by bronchodilation by inhalation of Salbutamol, 0.2 mg from spacer (AeroChamber Plus Flow-Vu) following the ERS guidelines for standardisation of spirometry [17].

### Diagnoses

Asthma at baseline was defined by an affirmative answer to the question ‘Have you ever had asthma’? combined with asthma-like symptoms, use of medication for breathing within the last 12 months or airflow obstruction according to a modified definition used by de Marco et al. in a recent study [18] (Table S1). Obstruction was defined according to the lower limit of normal (LLN) [19] i.e. the 5th percentile of FEV₁/FVC distribution corresponding to a z-score <−1.64. At baseline, the maximum values of FEV₁ and FVC were applied without reversibility testing since methacholine challenge test was performed. At follow up, the maximum value was the best of either the pre-bronchodilator (pre-BD) or post-bronchodilator (post-BD) value. COPD was defined according to criteria of LLN combined with symptoms consistent with COPD, modified from de Marco et al. [18] (Table S1). Transient airflow obstruction was defined by obstruction at baseline but no obstruction at follow up, while fixed obstruction was defined by having post-BD obstruction at follow up. Incident cases of asthma and COPD during the follow-up period were identified by applying the definitions used at baseline, although slightly modified since BHR was not measured at follow up (Table S1). Asthma-COPD overlap syndrome (ACOS) was defined when criteria for both asthma and for COPD were met. Early-onset asthma was defined when age of first attack of asthma was less than 16 years and late-onset when 16 years or more. Remission of asthma was defined by not fulfilling the criteria for asthma at follow up.

### Determinants

BHR at baseline was defined as a 20% fall or more in FEV₁ after a dose of 1 mg methacholine or less. The bronchodilation was positive with an increase in FEV₁ of ≥12% and ≥200 ml. FEV₁ below predicted was defined by FEV₁<100% predicted corresponding to a z-score<0 [19]. Atopy was defined by one or more positive SPT (mean wheal diameter ≥3 mm). The type of medication used for breathing within the last 12 months was recorded and categorised into three levels: (1) no medication, (2) only short-acting beta-adrenoceptor agonists (SABA), and (3) inhalation corticosteroid (ICS) and/or long-acting beta-adrenoceptor agonists (LABA). The applied five-item asthma score was developed by Pekkanen, Sunyer et al. [20,21] and consisted of the simple sum of affirmative answers to five main asthma-like symptoms ranging from zero to five, not including questions regarding asthma attacks or asthma medication, in order to grade symptoms of asthma as a continuum (Table S2). Current smoking at baseline was defined in individuals, who reported smoking for at least one year and were still smoking. Occupation at baseline, reported as the last held job, was coded according to the International Standard Classification of Occupations from 1988 (ISCO88) and classified in high- and low-risk jobs for asthma and COPD, respectively using job grouping tools formerly applied in the ECRHS [22]. Furthermore, participants were categorised in white and blue collar workers by using ISCO88-code <6000 and ≥6000, respectively.

### Statistical analyses

Univariate and multivariate analyses were conducted by logistic regression models calculating odds ratios (OR) with 95% confidence intervals (CI) for the association between the dependent outcome (asthma or COPD) and the independent determinants with mutual adjustment for potential confounders. Univariate analyses were performed on a comprehensive set of potential determinants. For the multivariate analyses, a reduced set of determinants was selected based on clinical relevance and specific interest i.e. sex, FEV₁ below pred., BHR, ACOS, asthma score, medication for breathing, current smoking, and high-risk occupation. The analyses of asthma score as an outcome were performed using the score as a continuous variable. Supplementary we analysed the asthma score as a categorical variable.

Asthma score was analysed by ordered logistic regression calculating ORs. FEV₁ at baseline and at follow up was analysed by linear regression. Results were considered statistically significant at *p* < 0.05. Analyses were carried out using Stata, version 13.1 (StataCorp, College Station, Texas, USA).

## Results

Among the 1,191 participants of the baseline study, 449 individuals were lost to follow up. Withdrawal analyses showed that the proportion of cases of asthma at baseline did not differ between participants and non-responders (32.2% vs. 27.8%, *p* = 0.120). Compared to non-responders a larger proportion of the participants were older than 35 years old (53.6% vs. 41.4%; *p* = 0.000) and reported nasal allergy (42.1% vs. 32.3%; *p* = 0.001) whereas a smaller proportion were female (53.6% vs. 60.6%; *p* = 0.022) and current smoker (27.0% vs. 33.0%; *p* = 0.030). Participants and non-responders did not differ significantly concerning the other variables analysed.

Among the 239 individuals with asthma at baseline, 193 (81%) had asthma solely while 46 (19%) had ACOS. During follow up, 68 (28%) individuals were in remission and seven (3%) were diagnosed with COPD without having asthma. This left 164 (69%) individuals with persistent asthma at follow up of whom 136 (83%) had asthma solely and 28 (17%) had ACOS. Individuals with ACOS at baseline had a lower remission rate than those with asthma solely (9% vs. 33%; *p* = 0.009).

Characteristics of the study population and associations with determinants at baseline by diagnosis at follow up are shown in Table 1. There was an almost equal distribution of individuals with early- and late-onset asthma. The risk of persistent asthma increased with increasing asthma score at baseline, both when the score was analysed as a continuous variable and when analysed as a categorical variable (data not shown). Current smoking at baseline was associated with a reduced risk of persistent asthma at follow up. Of 68 individuals who were smoking at baseline, 27 (40%) ceased smoking during follow up of whom 15 had persistent asthma and 12 were in remission at follow up (*p* = 0.88).

**Table 1.** Characteristics of the asthmatic individuals and unadjusted associations of baseline determinants (OR) with persistent asthma and COPD at follow up.

		Diagnosis at follow up			
	Asthma at baseline (*n* = 239)	Persistent asthma (*n* = 164)		COPD (*n* = 35)	
Determinants, baseline	*n* (%)	*n* (%)	OR (95%CI)	*n* (%)	OR (95%CI)
Sex, female	150 (62.8%)	99 (60.4%)	0.72 (0.38–1.32)	22 (62.9%)	1.00 (0.45–2.31)
Age ≥35 years	122 (51.1%)	80 (48.8%)	0.75 (0.42–1.34)	22 (62.9%)	1.76 (0.80–4.01)
Age of onset ≥16 years^a^	119 (55.6%)	81 (52.3%)	0.60 (0.31–1.17)	15 (48.4%)	0.71 (0.31–1.64)
Bronchial hyperresponsiveness^b^	95 (39.8%)	65 (39.6%)	1.11 (0.60–2.07)	14 (40.0%)	1.81 (0.71–4.81)
Atopy	146 (61.1%)	104 (63.4%)	1.36 (0.75–2.46)	17 (48.6%)	0.55 (0.25–1.21)
FEV₁ below pred	191 (79.9%)	139 (84.8%)	**2.46(1.21–4.95)**	33 (94.3%)	**4.80(1.15–42.65)**
ACOS	46 (19.3%)	39 (23.8%)	**3.03(1.25–8.43)**	19 (54.3%)	**7.78(3.31–18.23)**
Medication, 12 mth					
SABA only	63 (26.4%)	48 (29.3%)	**5.06(2.43–10.54)**	8 (22.9%)	1.15 (0.42–3.17)
ICS and/or LABA	96 (40.2%)	85 (51.8%)	**12.21(5.64–26.45)**	18 (51.4%)	1.82 (0.77–4.31)
Asthma Score^c^			**1.48(1.20–1.82)**		1.16 (0.91–1.48)
BMI >30 kg^g^m^−2^^d^	46 (19.3%)	31 (19.0%)	0.94 (0.45–2.02)	5 (14.3%)	0.66 (0.19–1.87)
Nasal allergy	168 (70.3%)	122 (74.4%)	1.83 (0.98–3.41)	22 (62.9%)	0.67 (0.30–1.56)
Parental asthma	92 (38.5%)	67 (40.9%)	1.38 (0.75–2.57)	13 (37.1%)	0.93 (0.41–2.07)
Current smoking	68 (28.5%)	37 (22.6%)	**0.41(0.22–0.78)**	14 (40.0%)	1.85 (0.81–4.13)
High-risk occupation (asthma)^e^	119 (50.4%)	81 (50.0%)	0.95 (0.53–1.70)	15 (42.9%)	0.70 (0.31–1.53)
High-risk occupation (COPD)^f^	126 (53.4%)	85 (52.5%)	0.89 (0.49–1.60)	16 (45.7%)	0.70 (0.32–1.52)
White collar work^g^	153 (64.6%)	103 (63.2%)	0.70 (0.32–1.47)	22 (62.9%)	0.76 (0.31–1.93)
Blue collar work^g^	55 (23.2%)	41 (25.2%)	1.42 (0.68–3.09)	10 (28.6%)	1.32 (0.52–3.18)
Respiratory infection <5 years of age	30 (12.6%)	23 (14.0%)	1.58 (0.62–4.58)	8 (22.9%)	2.45 (0.85–6.43)
Mould inside the home	85 (35.6%)	57 (34.8%)	0.89 (0.49–1.65)	15 (42.9%)	1.44 (0.64–3.15)

**Bold** values denote significant associations (*p* < 0.05). ^a^*n* = 214; 25 participants missing in variable ‘age at onset’. ^b^*n* = 210; 29 missing i.e. did not receive methacholine challenge test ^c^Asthma score as a continuous variable. ^d^*n* = 238; 1 participant missing in variable BMI. ^e^*n* = 236; 3 participants missing in variable ‘high-risk occupation (asthma)’. ^f^*n* = 236; 3 participants missing in variable ‘high-risk occupation (COPD)’. ^g^*n* = 208; 2 participants missing, 29 unclassifiable. The table shows odds ratios (OR) with 95% confidence intervals (95% CI).

**Figure 1.**
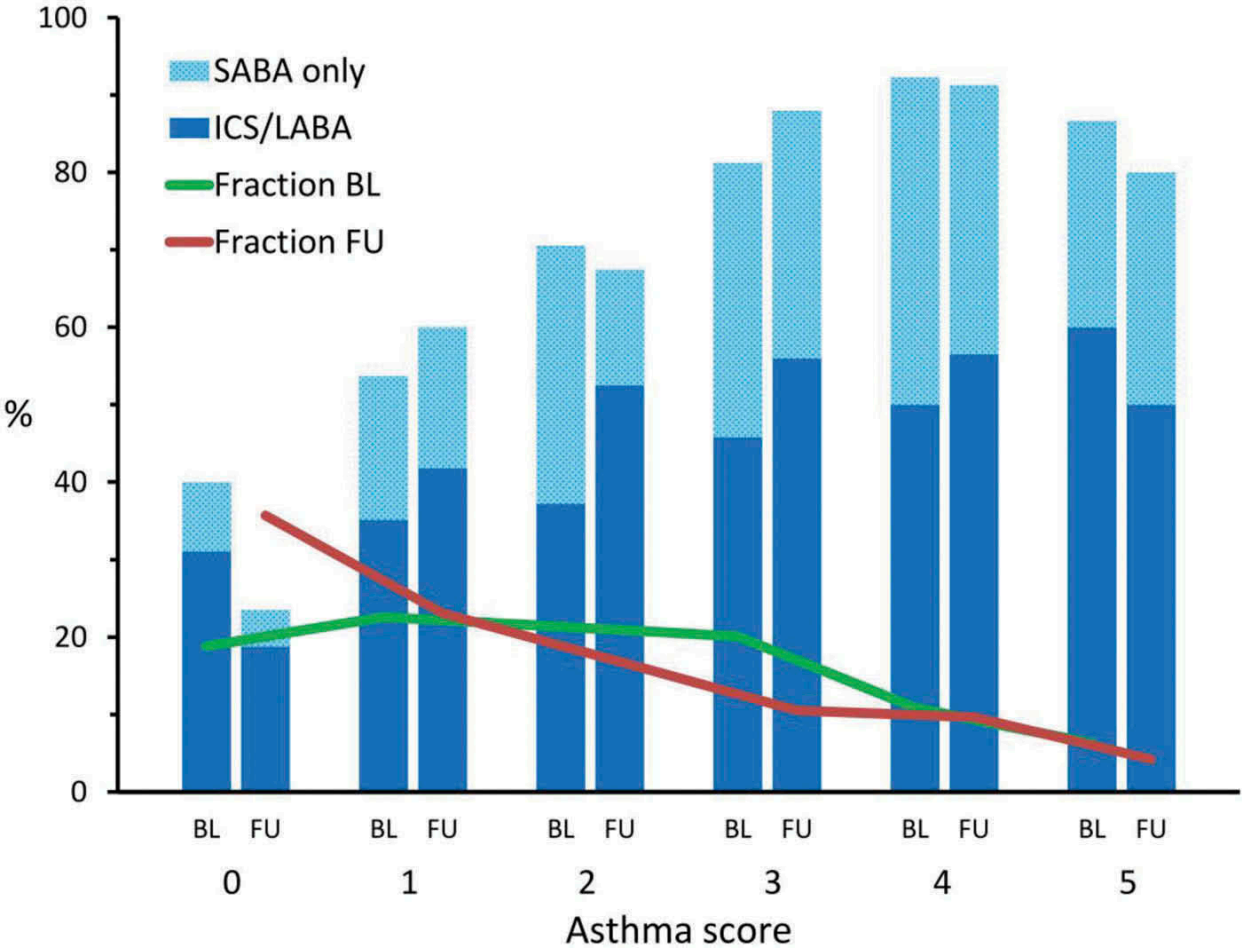
The distribution of asthma score at baseline (green) and follow up (red) and the percentage of individuals in each group reporting use of medication for breathing within the last 12 months by asthma score at baseline and follow up, respectively.

Figure 1 shows that the average five-item asthma score decreased from baseline to follow up (mean 2.00 vs. 1.47, *p* < 0.001), which was also the case for the group of individuals with persistent asthma (mean 2.25 vs. 1.86, *p* = 0.004). The percentage of individuals reporting use of medication for breathing increased with increasing asthma score, but there was no change in the proportion of use of medication from baseline to follow up.

All of the five questions defining asthma score predicted risk of persistent asthma in unadjusted analyses (Table S2), but only an affirmative answer to the question ‘shortness of breath while wheezing or whistling in the last 12 months’ revealed an increased risk of persistent asthma in the mutually adjusted analysis. None of the questions showed significant association to COPD at follow up.

In the adjusted analyses, determinants associated with persistence of asthma at follow up (Table 2) were use of SABA and use of ICS and/or LABA, whereas current smoking showed a reduced risk. Age of onset of asthma showed heterogeneity. In individuals with early-onset asthma FEV₁ below predicted, asthma score and use of ICS and/or LABA at baseline determined an increased risk, while use of ICS and/or LABA was the only determinant of persistent late-onset asthma.

**Table 2.** Determinants for persistent asthma and COPD at follow up by logistic regression in 239 individuals with asthma at baseline.

	Follow up					
	Persistent asthma, OR (95%CI)			COPD, OR (95%CI)		
Determinants, Baseline	All (*n* = 164)	Early-onset asthma (*n* = 74)^a^	Late-onset asthma (*n* = 81)^a^	All (*n* = 35)	Early-onset asthma (*n* = 16)^b^	Late-onset asthma (*n* = 15)^b^
Sex, female	0.70 (0.35–1.40)	0.69 (0.17–2.83)	0.70 (0.26–1.84)	1.43 (0.61–3.38)	**20.40(1.57–265.69)**	0.71 (0.15–3.33)
FEV₁ below pred	2.12 (0.98–4.61)	**7.12(1.61–31.50)**	1.53 (0.53–4.40)	3.05 (0.67–13.91)	2.91 (0.23–37.35)	NA
Bronchial hyperresponsiveness	1.01 (0.51–2.00)	2.18 (0.36–13.21)	1.13 (0.43–2.97)	0.79 (0.35–1.81)	**0.06(0.01–0.56)**	3.04 (0.68–13.70)
ACOS	2.60 (0.89–7.62)	2.81 (0.23–34.00)	1.27 (0.29–5.63)	**6.98(2.84–17.14)**	**204.96(9.20–4566.65)**	**15.53(3.22–74.76)**
Asthma Score per step increase.	1.21 (0.94–1.56)	**2.06(1.15–3.68)**	1.12 (0.80–1.56)	1.03 (0.78–1.37)	1.39 (0.81–2.39)	0.84 (0.52–1.34)
Medication, 12 mth						
SABA only	**3.39(1.47–7.82)**	4.52 (0.81–25.22)	2.26 (0.69–7.42)	0.90 (0.27–2.99)	5.06 (0.60–42.67)	0.25 (0.02–2.84)
ICS and/or LABA	**8.95(3.87–20.69)**	**9.87(1.95–49.98)**	**6.84(2.09–22.37)**	1.22 (0.41–3.59)	0.38 (0.05–3.19)	1.29 (0.19–8.67)
Current smoking	**0.45(0.21–0.94)**	1.01 (0.20–5.15)	0.98 (0.35–2.72)	1.86 (0.74–4.70)	4.54 (0.67–30.53)	2.25 (0.51–10.00)
High-risk occupation (asthma) ^c^	2.48 (0.45–13.72)	1.18 (0.04–33.77)	1.97 (0.26–14.90)	0.35 (0.03–3.59)	NA	0.08 (0.00–2.33)
High-risk occupation (COPD)^d^	0.53 (0.10–2.87)	1.25 (0.04–36.31)	0.57 (0.08–4.15)	1.93 (0.20–18.83)	NA	7.78 (0.31–197.17)

**Bold** values denote significant associations (*p* < 0.05). ^a^*n* (total) = 155 due to missing data on age at first asthma attack. ^b^*n* (total) = 31 due to missing data on age at first asthma attack. ^c^*n* = 236; 3 participants had missing in variable ‘high-risk occupation (asthma)’. ^d^*n* = 236; 3 participants had missing in variable ‘high-risk occupation (COPD)’. NA: Not applicable The table shows odds ratios (OR) with 95% confidence intervals (95% CI).

The adjusted analyses of the association between baseline determinants and asthma score at follow up and FEV₁ at baseline and follow up are shown in Table 3. At follow up, all determinants except current smoking were positively associated with asthma score although not significantly, which was independent of the diagnostic criteria applied.

**Table 3.** Association between baseline determinants and asthma score at follow up (OR per step increase), FEV₁ at baseline and FEV₁ at follow up (coeff.).

	Asthma score at follow up	FEV₁ at baseline (liter)	FEV₁ at follow up (liter)
Determinants, baseline	OR (95%CI)	Coefficient (95%CI)	Coefficient (95%CI)
Sex, female	1.07 (0.65–1.74)	−**1.006 (−1.144−0.868)**	0.050 (−0.050–0.151)
FEV₁	–	–	1.026 (0.958–1.095)
FEV₁ below pred.	1.44 (0.78–2.66)	–	–
Bronchial hyperresponsiveness	1.37 (0.84–2.23)	−0.014 (−0.149–0.121)	−0.004 (−0.075–0.068)
ACOS	1.66 (0.88–3.11)	**−0.398 (−0.576−0.221**)	0.025 (−0.072–0.123)
Asthma Score per step increase	**1.60 (1.34–1.91)**	0.007 (−0.042–0.055)	−0.001 (−0.027–0.024)
Medication, 12 mth			
SABA only	1.50 (0.77–2.94)	0.046 (−0.138–0.231)	**0.099 (0.002–0.196)**
ICS and/or LABA	1.14 (0.63–2.08)	0.005 (−0.162–0.172)	0.029 (−0.059–0.117)
Current smoking	0.89 (0.51–1.57)	−0.141 (−0.295–0.014)	0.020 (−0.062–0.102)
High-risk occupation (asthma)^a^	1.16 (0.31–4.28)	0.133 (−0.266–0.531)	0.076 (−0.134–0.285)
High-risk occupation (COPD) ^b^	1.26 (0.34–4.68)	−0.047 (−0.444–0.350)	−0.042 (−0.250–0.167)

**Bold** values denote significant associations (*p* < 0.05). ^a^*n* = 236; 3 participants had missing in variable ‘high-risk occupation (asthma)’. ^b^*n* = 236; 3 participants had missing in variable ‘high-risk occupation (COPD)’. Regarding FEV₁ negative numbers report larger decrease. The table shows odds ratios (OR) and regression coefficients with 95% confidence intervals (95% CI).

FEV₁ decreased in average 196 ml from baseline to follow up corresponding to 21.3 ml/year, which is 19.4 ml/year in individuals with early-onset asthma and 22.5 ml/year in late-onset (data not shown). At baseline, the presence of ACOS was associated with a reduced FEV₁. At follow up, only medication with SABA predicted a lower decline in FEV₁ compared to those without medication. If the initial FEV_1_ at follow up was analysed the average annual fall was 37.6 ml/year with no difference related to age at onset.

## Discussion

In the present population-based cohort study of young adults, a considerable remission rate was found and during the 9-year follow up the average asthma score based on frequency of asthma-like symptoms declined even in individuals with persistent asthma. Using medication for breathing within 12 months before baseline predicted overall persistent asthma nine years later. Age at onset of asthma showed different determinants of persistent asthma as FEV₁ below predicted, asthma score and use of ICS and/or LABA were all determinants in individuals with early-onset asthma, while use of ICS and/or LABA was the only determinant in individuals with late-onset asthma. However, there was indication of insufficient asthma control in the present cohort since a considerable fraction of individuals with persistent asthma had several symptoms at follow up.

The different baseline determinants associated with an increased risk of persistent asthma in the two groups described by the age of onset may reflect characteristics of two distinct phenotypes. We demonstrated an impact of FEV₁ below predicted on the risk of persistent asthma in individuals with early-onset asthma, which is in accordance with a recent study in which the impact on lung function of early-onset asthma was considerably greater than for late-onset asthma [23]. We were not able to show an impact of atopy on the persistence of asthma, but in a recent review comparing studies on early- and late-onset of current asthma, the findings showed that adults with early-onset disease were more likely to be atopic and had a higher frequency of asthma attacks, whereas adults with late-onset disease were more likely to be female and had greater degrees of fixed airflow obstruction [24].

The use of medication at baseline was a determinant for persistence of disease in the present study. The use of ICS and/or LABA was a stronger determinant than the use of SABA only. This may reflect more severe disease when using long-term controllers than short-term relievers. The strongest association was seen in individuals with early-onset asthma which may likewise indicate more severe disease. Use of asthma medication in early- and late-onset asthma has been reported, but details vary between studies [24].

In the present study, the applied asthma score demonstrated its usability since increasing asthma score at baseline predicted an increased risk of persistent asthma in individuals with early-onset asthma, while there was no increased risk of later COPD. A substantial proportion of individuals had asthma score equal to zero at baseline (18.8%) and at follow up (36.8%). This may be due to mild cases diagnosed by the diagnostic criteria or due to individuals who remitted during follow up. However, results concerning asthma score in the adjusted models must be interpreted with caution since the five questions comprising the asthma score are part of the diagnostic criteria, which additionally includes supplementary questions on asthma attacks and medication as well as BHR and airway obstruction. When the asthma score was analysed independently of the diagnostic criteria (Table 3), a positive association was still demonstrated in the majority of determinants analysed thus supporting the applicability of the score.

The decreased asthma score during follow up may reflect the individuals who achieved remission during follow up as well as regression towards the mean since all individuals had asthma at baseline. Alternatively, it may imply some effect of asthma treatment during follow up or that the asthma score actually does not fully cover the spectrum of symptoms. However, at follow up a substantial part of individuals who reported use of medication for breathing had a considerable number of symptoms expressed by the asthma score indicating partly controlled or uncontrolled asthma. This emphasises the need for further medication even though some may have treatment resistant asthma.

We showed no overall change in use of medication for breathing from baseline to follow up. A considerable proportion of individuals were not medicated at all regardless of symptoms and even in individuals with asthma scores on 4–5 more than 10% were not currently medicated and only about half used controller medication indicating a need for treatment even though poor adherence may also play a role [25]. These findings are in accordance with previous studies showing that insufficient symptom control of asthma remains frequent among individuals with asthma [26,27].

The lung function data showed an overall decline in FEV₁ of 21.3 ml per year in individuals with current asthma at baseline. This is not different from predicted in non-asthmatic subjects but the results may be evaluated with caution since pre-BD FEV₁ was used at baseline and post-BD at follow up which may tend to underestimate the value although best FEV₁ was used at both occasions. Analyses using pre-BD values showed larger decline per year, but did not influence on the role of the other determinants in the multivariate analyses. Previous studies have suggested accelerated lung function declines in asthma [28,29] and in a review of adult-onset asthma decline in FEV₁ varied between 25 and 95 ml per year [6]. Still, recent studies have found decline in FEV₁ in asthmatics of 25.3 [18] and 25.6 ml [30] per year, respectively. When adjusted for FEV₁ at baseline, determinants for change in lung function during follow up showed a smaller decrease of 50 ml (corresponding to 5.5 ml per year) in females than in males which may reflect the more than one liter lower FEV₁ in females overall compared to males at baseline. Equal to this, individuals with ACOS and individuals who were current smokers showed a reduced lung function of nearly 0.40 and 0.14 l, respectively, at baseline in comparison with their references i.e. healthy individuals and non-smokers, respectively. These determinants may have played a role even before baseline which may be the reason for the lowered decline in lung function than their references during follow up.

The issue of asthma and smoking remains controversial. In the present study, smoking was associated with a reduced risk of persistent asthma, which could be due to a ‘healthy smoker effect’, i.e. individuals with asthma at baseline had quit smoking earlier or had never started smoking due to airway symptoms since no difference between persistent asthma and remission was found among the individuals who ceased smoking during follow up. Previous studies have suggested that smoking may have a negative effect on longitudinal changes in lung function in individuals with asthma [31]. A recent large study on asthma in the general population aged 20–100 years showed that smoking was the main explanation of poor prognosis and comorbidities in individuals with asthma during 4.5 years of follow up [32].

We were not able to confirm the findings in other studies showing poor prognosis of asthma in individuals with high-risk occupation [33]. The analyses showed no significant correlation between high-risk occupation and persisting asthma, which could be due to the young age of the study population, the relatively short duration of follow up or a ’healthy worker effect’, i.e. that subjects with asthma before baseline had chosen an occupation that would not provoke or exacerbate their airway symptoms.

We found that 29% of the individuals with current asthma at baseline achieved remission during follow up, which is slightly higher than the recent comparable longitudinal study using similar follow-up period and diagnostic criteria in which the remission of current adult asthma was 22.2% [18]. Nearly the same range of remission was reported in a study of individuals with self-reported current asthma sampled from the Italian population in which 30% recovered from their asthma after about 10 years [34]. The prevalence of remission of asthma in adults has been reported in the range from 5 to 40%, and usually limited to individuals with mild disease [10,35,36]. However, remission rates can be difficult to compare since there is no golden standard to define remission [6].

Individuals with ACOS at baseline had higher – however not significant – risk of persistent asthma than individuals with asthma solely. This is in line with a recent study of young adults in the same age range [18] suggesting that ACOS may represent a phenotype with severe asthma, which progresses to fixed airflow obstruction, possibly due to structural changes in the airways. This is supported by the observation that the determinants for persistent asthma, i.e. use of medication were not associated to COPD at follow up. A recent study of different phenotypes of chronic airway diseases in the general population confirmed the poor prognosis of individuals with ACOS, especially in a subgroup with late-onset asthma defined by current self-reported asthma with onset after 40 years of age [30].

The present study is population-based which constitutes a strength when evaluating determinants for prognosis of asthma in the general population. The longitudinal study design and the use of validated and internationally applied questionnaires and clinical examinations including measurements of pulmonary function of all participants are further strengths [37,38]. By use of multivariate logistic regression models in the analysis, we believe to have controlled for confounding factors potentially able to have an impact on the outcome, i.e. persistent asthma.

Although the study was population based, a risk of selection bias exists since asthmatic individuals with more severe airway symptoms may be more prone to participate than individuals with minor symptoms. Misclassification of disease status may have occurred due to the choice of diagnostic criteria. By use of self-reported information, individuals in remission who reported ever asthma at baseline may have been mild cases without symptoms at follow up. Furthermore, we may have overestimated the number of cases of COPD by using the LLN by the 5th percentile of FEV₁/FVC corresponding to z-score <−1.64, and the relatively low dose of beta-agonist chosen due to the study setting in a general population [39]. The use of two different kinds of spirometers, one with a turbine (MicroLoop) at baseline and one with ultra-sound transit time measurement (ndd EasyOne) at follow up may have affected the lung function data although calibration check was performed daily on both spirometers to minimise this bias. Furthermore, definitions of the age limit between early- and late-onset asthma vary widely in the literature [6,8,24]. However, the applied criteria for asthma and COPD are in line with recent research [18]. The cut-off at 16 years was chosen at baseline in order to evaluate risk of occupational factors in a relevant group.

Limitations of the study include the size of the study population with a limited number of individuals having asthma leading to low power of the analyses even though we used an enriched sample, and further subgroup analyses to evaluate the impact of potential effect modification were not performed.

## Conclusion

The present study showed that determinants for persistent asthma in young adults differed according to age at onset of disease. Pulmonary function below predicted, increased asthma score and use of medication for breathing within 12 months before baseline determined persistence of asthma in individuals with early-onset disease, while use of medication was the only determinant for persistence in individuals with late-onset asthma. Use of controller medication for breathing showed stronger association with persistent asthma than only use of reliever medication in both groups.

Evaluation of asthma score and use of asthma medication indicated insufficiently treated asthma underlining the importance of regular monitoring of symptoms, pulmonary function, and treatment of adult asthma. Furthermore, in comparison with asthma, the diagnosis of ACOS at baseline was associated with an increased yet not significant risk of persisting asthma at follow up, which in line with other studies indicates that ACOS represents a phenotype of severe asthma.
